# Difference in Analgesic Effects of Repetitive Transcranial Magnetic Stimulation According to the Site of Pain

**DOI:** 10.3389/fnhum.2021.786225

**Published:** 2021-11-26

**Authors:** Nobuhiko Mori, Koichi Hosomi, Asaya Nishi, Dong Dong, Takufumi Yanagisawa, Hui Ming Khoo, Naoki Tani, Satoru Oshino, Youichi Saitoh, Haruhiko Kishima

**Affiliations:** ^1^Department of Neurosurgery, Osaka University Graduate School of Medicine, Suita, Japan; ^2^Department of Mechanical Science and Bioengineering, Osaka University Graduate School of Engineering Science, Toyonaka, Japan; ^3^Osaka University Institute for Advanced Co-Creation Studies, Suita, Japan; ^4^Tokuyukai Rehabilitation Clinic, Toyonaka, Japan

**Keywords:** repetitive transcranial magnetic stimulation (rTMS), motor cortex stimulation, neuropathic pain, meta-analysis, pain sites, upper limb, lower limb

## Abstract

High-frequency repetitive transcranial magnetic stimulation (rTMS) of the primary motor cortex for neuropathic pain has been shown to be effective, according to systematic reviews and therapeutic guidelines. However, our large, rigorous, investigator-initiated, registration-directed clinical trial failed to show a positive primary outcome, and its subgroup analysis suggested that the analgesic effect varied according to the site of pain. The aim of this study was to investigate the differences in analgesic effects of rTMS for neuropathic pain between different pain sites by reviewing our previous clinical trials. We included three clinical trials in this mini meta-analysis: a multicenter randomized controlled trial at seven hospitals (*N* = 64), an investigator-initiated registration-directed clinical trial at three hospitals (*N* = 142), and an exploratory clinical trial examining different stimulation parameters (*N* = 22). The primary efficacy endpoint (change in pain scale) was extracted for each patient group with pain in the face, upper limb, or lower limb, and a meta-analysis of the efficacy of active rTMS against sham stimulation was performed. Standardized mean difference (SMD) with 95% confidence interval (CI) was calculated for pain change using a random-effects model. The analgesic effect of rTMS for upper limb pain was favorable (SMD = −0.45, 95% CI: −0.77 to −0.13). In contrast, rTMS did not produce significant pain relief on lower limb pain (SMD = 0.04, 95% CI: −0.33 to 0.41) or face (SMD = −0.24, 95% CI: −1.59 to 1.12). In conclusion, these findings suggest that rTMS provides analgesic effects in patients with neuropathic pain in the upper limb, but not in the lower limb or face, under the conditions of previous clinical trials. Owing to the main limitation of small number of studies included, many aspects should be clarified by further research and high-quality studies in these patients.

## Introduction

Migita et al. (1995) reported the pain-relieving effects of repetitive transcranial magnetic stimulation (rTMS) in two patients with central neuropathic pain. Since then, rTMS has been developed as a promising, safe, and non-invasive brain stimulation treatment tool with fewer side effects for chronic pain that may benefit patients who do not respond to conventional pharmacological therapies. In the early 2000s, 10-Hz rTMS to the primary motor cortex (M1) was shown to be effective in patients with neuropathic pain (Lefaucheur et al., 2001a,b). Since then, many studies have been conducted to investigate the optimal parameters. M1 of the hand area contralateral to the painful side was stimulated regardless of the pain site in some previous studies (Lefaucheur et al., 2001a,b, 2008; Khedr et al., 2005, 2015; André-Obadia et al., 2006, 2008, 2011, 2021; Sun et al., 2019; Quesada et al., 2020). Lefaucheur et al. included patients with unilateral pain predominating at the hands because anatomically, the M1 of the hand area can be identified more reliably as the stimulation site than the face and lower limbs areas (Lefaucheur et al., 2001a, 2006a). Some other study groups restricted their inclusion criteria to predominantly upper limb pain patients with central post-stroke pain or complex regional pain syndrome, and they also showed that active TMS relieved pain more effectively compared with sham stimulation (Pleger et al., 2004; Picarelli et al., 2010; Ojala et al., 2021). In contrast, some studies have examined the effectiveness of rTMS in alleviating pain at different stimulation sites (Hirayama et al., 2006; Lefaucheur et al., 2006b; Jette et al., 2013; de Oliveira et al., 2014; Lindholm et al., 2015; Ayache et al., 2016; Nurmikko et al., 2016; André-Obadia et al., 2018; Galhardoni et al., 2019; Attal et al., 2021; Freigang et al., 2021; Ojala et al., 2021). We have reported that 5 Hz-rTMS to M1 relieved neuropathic pain, but that to the primary somatosensory cortex, premotor area, and supplementary motor area did not (Hirayama et al., 2006). Based on earlier promising results, we subsequently conducted a large, rigorous, multicenter, randomized, blinded, controlled, parallel trial involving 144 patients with neuropathic pain. The results showed that five daily sessions of rTMS over M1 with 500 pulses/session at 5 Hz did not achieve better pain relief than sham stimulation. However, the subgroup analysis suggested that the analgesic effect of rTMS for upper limb pain was superior to that of rTMS for lower limb pain (Hosomi et al., 2020). Similarly, another study reported that the efficacy of rTMS for upper limb pain tended to be higher than that for lower limb pain (Hosomi et al., 2013). Considering our previous studies, we hypothesized that the pain relief effects of rTMS might differ, depending on the pain site.

In some systematic reviews and therapeutic guidelines, high-frequency rTMS of the M1 for neuropathic pain has been shown to be effective (Jin et al., 2015; Cruccu et al., 2016; Singer et al., 2017; O’Connell et al., 2018; Aamir et al., 2020; Lefaucheur et al., 2020; Leung et al., 2020; Moisset et al., 2020; Shen et al., 2020; Yang and Chang, 2020; Attia et al., 2021; Knotkova et al., 2021; Zhang et al., 2021). Moreover, the effectiveness of high-frequency rTMS has also been reported in some types of painful conditions, such as various non-neuropathic pain (Galhardoni et al., 2015; Lan et al., 2017; Cardinal et al., 2019; Ferreira et al., 2019). In addition to reports on the efficacy of rTMS for a variety of pain-causing conditions, differences in the pain-relieving effects of rTMS have been investigated for a variety of factors, including stimulation location and frequency, number of stimulation pulses per session, and number of sessions. Although various factors that influence the pain-relieving effects of rTMS treatment have been investigated, evidence of the pain-relieving effects of rTMS by pain site is lacking. Therefore, the primary purpose of this study was to investigate the differences in analgesic effects of rTMS over M1 for neuropathic pain between different pain sites by reviewing our previous clinical trials.

## Materials and Methods

### Study Design, Studies Selection, and Data Source

This study was a meta-analysis based on the results of our previous studies, which aimed to investigate the pain-relieving effects of rTMS treatment by pain site. Meta-analyses must generally be performed according to a predetermined procedure, which is the preferred reporting item for systematic reviews and meta-analyses statements (Liberati et al., 2009; Rethlefsen et al., 2021). However, to the best of our knowledge, only two studies have examined the differences in the pain relief effects of high-frequency rTMS by pain site (Lefaucheur et al., 2004; Ayache et al., 2016). In these studies, the level of significance (*P*-value) is clearly provided, but the amount or rate of the decrease in pain intensity is not, and there is a lack of data available for meta-analysis by pain site. Therefore, we defined this study as a mini meta-analysis, because only our previous trials were extracted and analyzed. We extracted randomized controlled trials (RCTs) of rTMS using the figure-of-8 coil for neuropathic pain, conducted at Osaka University Hospital as the main study institution, because this was the first attempt to review the analgesic effects at different pain sites. We included three of our previous clinical trials in this meta-analysis (Hosomi et al., 2013, 2020; Mori et al., 2021b). Hosomi et al. (2013) conducted a randomized, double-blind, sham-controlled trial, from 2009 to 2011, at seven centers in Japan, to assess the efficacy and safety of 10 daily doses of rTMS in patients with neuropathic pain. A series of 10 daily 5-Hz rTMS (500 pulses/session) of M1 or sham stimulation was applied to each patient with a follow-up of 17 days. This study was divided into two groups: group A had an active rTMS period followed by a sham period, and group B had a sham period followed by an active rTMS period. Therefore, the two groups were analyzed separately in this analysis. From the data from Hosomi et al. (2013), we used the mean visual analog scale (VAS) decrease over 10 sessions for this analysis. This was calculated by subtracting the VAS value immediately before the intervention from the VAS value immediately after the intervention for each session and then averaging them. Second, in a trial by Hosomi et al. (2020), a randomized, patient- and assessor-blinded, sham-controlled, parallel trial was conducted from 2016 to 2017 at three centers to obtain regulatory approval in Japan. A series of five daily 5-Hz rTMS (500 pulses/session) of M1 or sham stimulation was applied to each patient with a follow-up of 4 weeks. We used the mean VAS decrease over five sessions for this analysis from the data of Hosomi et al. (2020). The mean decrease in VAS score was calculated using the procedure described by Hosomi et al. (2013). Finally, in a trial by Mori et al. (2021b), a randomized, single-blind, sham-controlled, crossover exploratory study was conducted from 2017 to 2018 at Osaka University Hospital to explore the optimal stimulus conditions for treating neuropathic pain. Four single sessions of M1-rTMS at different parameters (1, 5-Hz with 500 pulses per session; 2, 10-Hz with 500 pulses per session; 3, 10-Hz with 2000 pulses per session; and 4, sham stimulation) were conducted in random order. From the data of Mori et al. (2021b), we used VAS decrease, which was calculated by subtracting the VAS score immediately after the intervention from that immediately before the intervention for this analysis. Since Mori et al. (2021b) conducted a crossover study examining four different stimulation conditions, the results of the rTMS condition (10 Hz over M1 hand, 2000 pulses/session) that produced significantly more effective pain relief compared with the sham stimulation were extracted and integrated into the present study. These studies were approved by the institutional review boards, and written informed consent was obtained from all participants.

### Data Synthesis and Analysis

Changes in pain scale (VAS scale: 0 = no pain to 100 = maximal pain) were extracted as a primary efficacy endpoint from each trial, and a mini meta-analysis of the efficacy of active rTMS against sham stimulation was performed. Next, the efficacy of rTMS was analyzed for each patient group with pain in the face and upper or lower limbs. The chi-squared test and I^2^ statistic were used to quantify the heterogeneity between the trials. Heterogeneity was considered significant when chi-squared *P* < 0.10, and the I^2^ statistic was used to evaluate the degree of heterogeneity. Substantial heterogeneity was considered to be present when I^2^ was >50%. In this analysis, a random-effects model was used regardless of heterogeneity, considering the small sample size (Cochrane Handbook for Systematic Reviews of Interventions, Version 6.1, 2020; Chapter 10. Analyzing data and undertaking meta-analyses^1^). Standardized mean difference (SMD) with 95% confidence interval (CI) was calculated for pain change using a random-effects model. This analysis was performed using the Cochrane Collaboration’s software program Review Manager (RevMan) version 5.4.1. software (Cochrane Collaboration, Oxford, United Kingdom).

### Assessment of Resting Motor Threshold

The relationship between resting motor threshold (RMT) and pain site was examined in an investigator-initiated registration-directed clinical trial (Hosomi et al., 2020), in which RMT was recorded at M1 corresponding to the painful body part. The multicenter RCT (Hosomi et al., 2013) and the exploratory clinical trial (Mori et al., 2021b) were excluded from the RMT analysis because RMT was only partially recorded in the former, and RMT at M1 of the hand was recorded regardless of the pain site in the latter. The difference in RMT by pain site was analyzed using a one-way analysis of variance (ANOVA).

## Results

A total of 228 patients from three clinical trials were included in the analysis (Table 1). In group A of Hosomi et al. (2013), a total of 28 patients were included (upper limb pain, *n* = 15; lower limb pain, *n* = 8; facial pain, *n* = 5). The etiologies of neuropathic pain were as follows: cerebral lesion in 21 patients, spinal lesion in 4, peripheral nerve injury in 1, phantom limb in 1, and root avulsion in 1 patient. In group B of Hosomi et al. (2013), a total of 35 patients were included (upper limb pain, *n* = 20; lower limb pain, *n* = 14; facial pain, *n* = 1). The etiologies of neuropathic pain were as follows: cerebral lesion, 30 patients; spinal lesion, 3 patients; and phantom limb, 2 patients. In Hosomi et al. (2020), 142 patients were included (upper limb pain, *n* = 67; lower limb pain, *n* = 59; facial pain, *n* = 16). The etiologies of neuropathic pain were as follows: cerebral lesion, 54 patients; postherpetic neuralgia, 12 patients; spinal lesion, 9 patients; root avulsion, 9 patients; complex regional pain syndrome, 4 patients; phantom limb, 2 patients; and other lesions, 52 patients. In Mori et al. (2021b), a total 22 patients were included (upper limb pain, *n* = 10; lower limb pain, *n* = 10; facial pain, *n* = 2). The etiologies of neuropathic pain were as follows: cerebral lesion in 15 patients, complex regional pain syndrome in 3, peripheral nerve injury in 2, spinal lesion in 1, and root avulsion in 1 patient.

**TABLE 1:** Characteristics of our previous rTMS studies using the figure-of-eight coil.

Study	N	Pain origin (N)	Target of stimulation	Parameters and Dosage	Design/Study center	Stimulator/Coil	Sham condition
Hosomi et al., 2013	29	Stroke (22), Spinal lesion (4), Phantom limb (1), Root avulsion (1), Peripheral nerve injury (1)	M1 contralateral to painful side	5-Hz, 90%RMT, total 500 pulses/session (50 pulses × 10 train/session, 10 sessions, ITI = 50 s)	Cross-over RCT/7 centers	Magstim Rapid, Magstim Company, or AAA- 81077, Nihon Kohden Corp./figure-of-8	Sham coil + simultaneous electrical stimulation to the scalp
Hosomi et al., 2013	35	Stroke (30), Spinal lesion (3), Phantom limb (2), Root avulsion (0), Peripheral nerve injury (0)					
Hosomi et al., 2020	Active: 72	Stroke (31), Spinal lesion (2), Postherpetic neuralgia (6), Root avulsion (4), Phantom limb (2), CRPS (2), Other (25)	M1 contralateral to painful side	5-Hz, 90%RMT, total 500 pulses/session (50 pulses × 10 train/session, 5 sessions, ITI = 50 s)	Parallel RCT/3 centers	TEN-P11, Teijin Pharma Limited/eccentric figure-of-8	
	Sham: 70	Stroke (23), Spinal lesion (7), Postherpetic neuralgia (6), Root avulsion (5), Phantom limb (0), CRPS (2), Other (27)					Sham coil + simultaneous electrical stimulation to the scalp
Mori et al., 2021b	22	Stroke (15), CRPS (3), Spinal lesion (1), Root avulsion (1), Peripheral nerve injury (2)	the M1 hand area contralateral to the painful side	10-Hz, 90%RMT, total 2000 pulses/session (50 pulses × 40 train/session, 1 session, ITI = 25 s)	Cross-over RCT/single center	MagPro X100, MagVenture/figure-of-8	Sham coil

*N, Numbers of subjects; RMT, Resting Motor Threshold; M1, Primary motor cortex; CRPS, Complex Regional Painful Syndrome; RCT, Randomized Controlled Trial; ITI, Inter-train Interval; sec, second.*

*The Hosomi et al. (2013) (treatment group A) study includes participants with an active rTMS period followed by a sham period, and the Hosomi et al. (2013) (treatment group B) study includes participants with a sham period followed by an active rTMS period.*

**FIGURE 1:**
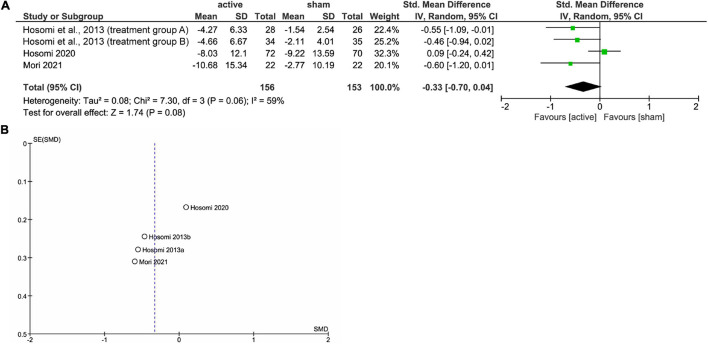
The forest plot **(A)** and funnel plot **(B)** of the analysis for the whole population.

**FIGURE 2:**
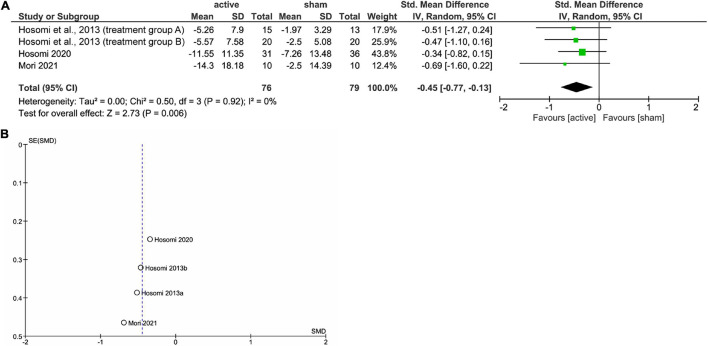
The forest plot **(A)** and funnel plot **(B)** of the analysis for the upper limb pain.

**FIGURE 3:**
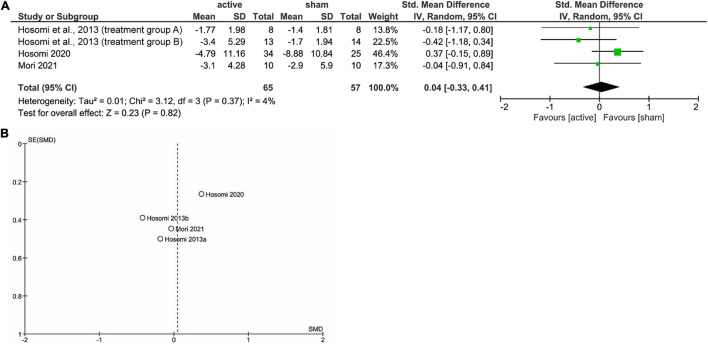
The forest plot **(A)** and funnel plot **(B)** of the analysis for the lower limb pain.

**FIGURE 4:**
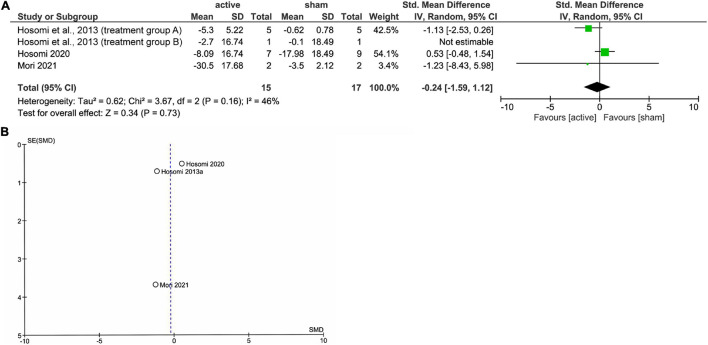
The forest plot **(A)** and funnel plot **(B)** of the analysis for the facial pain.

Figure 1 shows the results of the mini meta-analysis for the entire population. Heterogeneity was moderate (Chi-squared = 7.30, *P* = 0.06, *I*^2^ = 59%), and the analysis of the pooled analgesic outcome showed that the effect size was not statistically significant −0.33 (95% CI, −0.70 to 0.04; *P* = 0.08). In the analysis of the group of upper limb pain, heterogeneity was low (Chi-squared = 0.50, *P* = 0.92, *I*^2^ = 0%), and the analysis of the pooled analgesic outcome showed a significant effect size of −0.45 (95% CI, −0.77 −−0.13; *P* < 0.01) (Figure 2). This suggests that rTMS for neuropathic pain in the upper limbs was effective in decreasing pain intensity. In the analysis of the groups of lower limb pain and facial pain, heterogeneity was low (Chi-squared = 3.12, *P* = 0.37, *I*^2^ = 4%) and moderate (Chi-squared = 3.67, *P* = 0.16, *I*^2^ = 46%), respectively. The effect size for pain change was 0.04 (95% CI, −0.33 to 0.41; *P* = 0.82) for lower limb pain and −0.24 (95% CI, −1.59 to 1.12; *P* = 0.73) for facial pain (Figures 3, 4). rTMS was unlikely to be effective for neuropathic pain in the lower limbs or faces. The funnel plots were symmetrical, suggesting that the publication bias was low.

**FIGURE 5:**
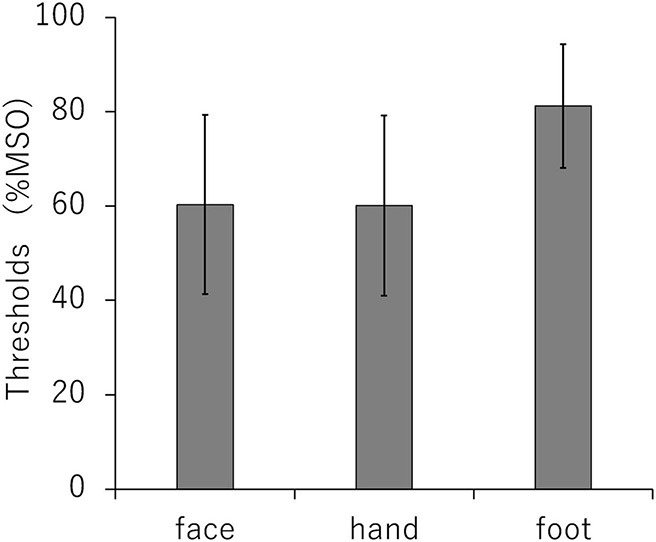
Comparison of resting motor thresholds for each stimulation site (primary motor cortex face, hand, and foot area). MSO, maximum stimulus output.

According to Hosomi et al. (2020), the mean RMTs (SD) at M1 face, hand, and foot were 60.3% of the maximum stimulator output (19.0), 60.1% (19.1), and 81.2% (13.1), respectively. There was a significant difference in RMT by pain site (*P* < 0.01) (Figure 5).

## Discussion

This study investigated the differences in analgesic effects of rTMS over the M1 using a figure-of-8 coil for neuropathic pain between pain sites. The findings from the three extracted clinical trials showed that rTMS for patients with neuropathic pain in the upper limb was particularly effective. Meanwhile, rTMS for patients with neuropathic pain in the lower limb and face was not confirmed to be effective.

Our main finding showed that high-frequency rTMS treatment using a figure-of-8 coil for neuropathic pain (*N* = 226) had a different effect on pain relief at the pain site. There have been many studies on the pain relief effects of rTMS for neuropathic pain, but to the best of our knowledge, there are few reports on the differences in pain relief effects by pain site (Lefaucheur et al., 2004; Ayache et al., 2016). Ayache et al. showed that pain intensity was significantly reduced after rTMS for both upper and lower limb pain (*N* = 20 and 16, respectively) (Ayache et al., 2016). Lefaucheur et al. showed that rTMS was more effective over the M1 hand area in facial pain (*N* = 14) than in upper and lower limb pain (*N* = 27 and 19, respectively) (Lefaucheur et al., 2004). The participants in these studies experienced a mixed condition of neuropathic pain, that is, central or peripheral neuropathic pain, similar to the participants in our three clinical trials. The stimulation condition set by Hosomi et al. (2013, 2020) was relatively low compared to those set by Lefaucheur et al. (2004) and Ayache et al. (2016) (5 Hz with 500 pulses/session vs. 10 Hz with 1,000 to 3,000 pulses/session). Mori et al. (2021b) adopted a high-dose (10 Hz with 2,000 pulses/session), but the results were not favorable for lower limb pain (SMD −0.04, 95% CI −0.91 to 0.84). The other difference was the manner of sham stimulation. In our previous trials, realistic sham stimulation, which produces scalp sensations and sounds similar to active stimulation without cortical stimulation, was performed by electrical stimulation and kept the conditions as similar as possible between active and sham stimulation. Consequently, realistic sham stimulation may have produced a large placebo effect. Although the manner of sham stimulation has differed between clinical trials, we do not think it is related to the difference in pain relief effect by pain site. Thus, the following comparison assessed the differences between the meta-analysis of the Cochrane review in single-session studies of high-frequency rTMS of M1 and this study. The SMD (95% CI) of pain score change in the Cochrane review (*N* = 249) was −0.38 (−0.49 to −0.27), and that for the whole population in this study was −0.33 (−0.70 to 0.04). Furthermore, the SMD (95% CI) for upper limbs pain in this study was −0.45 (−0.77 to −0.13). The SMDs (95% CI) of the two meta-analyses did not seem to be significantly different. Therefore, the results of the current study suggest that rTMS is clearly effective for upper limb pain and less effective for lower limb pain, as far as our previous studies are concerned.

We need to consider the factors that contribute to the difference in pain relief effects of rTMS for upper and lower limb pain. First, we showed that the RMT of the hand was clearly lower than that of the lower limb (Hosomi et al., 2013, 2020; Shimizu et al., 2017). Previous studies have demonstrated the correlation between RMT and the distance from the coil to the superficial layer of the brain (Stokes et al., 2005; Shimizu et al., 2017). Furthermore, previous studies in healthy subjects have reported that RMT was higher in the lower limb muscle (tibialis anterior) than that in the upper limb muscle (first dorsal interosseous), and it was higher in the figure-of-8 coil than that in the double-cone coil. The magnetic field strength produced by the double-cone coil is higher than that of the figure-of-8 coil (Schecklmann et al., 2020). This is due to the fact that the electric field generated by the figure-of-8 coil attenuates in relation to the depth of the target. Therefore, it is difficult to sufficiently stimulate the deep part of the brain with the figure-of-8 coil, especially the M1 foot area. To solve this problem of insufficient stimulation of the M1 foot area, rTMS with 3,000 pulses per session was performed under different stimulation conditions, such as stimulation intensity (90 or 110% RMT), coil position (M1 hand or foot area), and coil direction (anteroposterior or mediolateral) (Mori et al., 2021a). The results indicated that the analgesic effect was obtained in all conditions except sham stimulation, but simply increasing the intensity of the stimulation was not enough to eliminate pain. Second, the H-coil and double-cone coil, which can generate electric fields in the deep brain, have been used to stimulate the deep brain more effective than the figure-of-8 coil. One study investigated the analgesic effects of rTMS over the anterior cingulate cortex using an H-coil (Galhardoni et al., 2019). In addition, there are also reports that investigated the analgesic effect on foot pain with rTMS targeting the M1 foot using the H-coil (Onesti et al., 2013; Shimizu et al., 2017). In contrast, a pilot study of rTMS using a double-cone coil for chronic lower limb pain and a circular coil for upper limb pain failed to show significantly more effective pain relief compared with sham stimulation (Rollnik et al., 2002). According to a case report of invasive motor cortical stimulation, electrodes were implanted in the epidural area of the cortical regions corresponding to the painful area in patients with pain in the upper and lower limbs (Pommier et al., 2020). The stimulation of lower limb pain was inadvertently turned off and did not produce sufficient analgesic effect, but a turned-on stimulation reproduced sufficient pain relief. In addition, we implanted the subdural electrode over M1 corresponding to the painful site (Saitoh et al., 2000; Hosomi et al., 2008). Electrodes were implanted in the midline of the brain surface or in the interhemispheric fissure for lower limb pain (Hosomi et al., 2008). Presumably, another group used a similar technique to implant electrodes (Nguyen et al., 2011). These findings indicate the somatotopically driven analgesic efficacy of neuromodulation for lower limb pain. To alleviate pain with rTMS, it may be necessary to properly stimulate the target region. In the future, the efficacy of rTMS using an H-coil or double-cone coil for chronic pain needs to be further investigated.

In this meta-analysis, we focused on differences in the analgesic effects of rTMS over M1 at different pain sites. To date, some RCTs have been conducted in many centers to investigate various conditions. For example, trials have examined the pain-relieving effects of rTMS at different frequencies, different stimulation sites, and in single or multiple sessions, as well as trials examining the efficacy of rTMS for various neuropathic pain conditions, such as spinal cord injury (Yilmaz et al., 2014; Nardone et al., 2017; Sun et al., 2019), phantom limb pain (Ahmed et al., 2011; Malavera et al., 2016), traumatic brain injury (Choi et al., 2018), and radiculopathy (Attal et al., 2016). In recent years, some systematic reviews and meta-analyses have shown the efficacy and stimulation parameters of high-frequency rTMS for neuropathic pain (Baptista et al., 2019; Lefaucheur et al., 2020; Leung et al., 2020), and a practical algorithm for rTMS in the treatment of chronic pain in daily clinical practice has been proposed (Lefaucheur et al., 2020). In this algorithm, the stimulation site is not set to one specific area but to the M1 of the hand contralateral to the painful side or to the M1 corresponding to the painful area. If no improvement in pain is obtained, flexible parameters are proposed to change to a different stimulation site. Although this algorithm is a good clinically adapted setting, we consider that the optimal stimulation site to produce the analgesic effects of rTMS is controversial. Therefore, we reviewed RCTs of high-frequency rTMS in more than 10 patients with neuropathic pain (duration of more than 3 months) (Supplementary Table 1). We found that the M1 lower limb region was stimulated for lower limb pain in two studies using an H-coil (Onesti et al., 2013; Shimizu et al., 2017) and nine studies (Hirayama et al., 2006; Defrin et al., 2007; Saitoh et al., 2007; Kang et al., 2009; Hosomi et al., 2013, 2020; Jette et al., 2013; Ayache et al., 2016; Nurmikko et al., 2016) using a figure-of-8 coil. In contrast, the stimulation site in more than 10 studies targeted the M1 hand area, regardless of the pain site. A recent large RCT reported that rTMS over the M1 hand area was effective in patients with peripheral neuropathic pain. Approximately 60% of the participants were patients with lower limb pain (Attal et al., 2021). Previous studies have reported the efficacy of rTMS for peripheral neuropathic pain (Lefaucheur et al., 2004; Attal et al., 2016; Pei et al., 2019), and rTMS may be effective for peripheral neuropathic pain regardless of the pain site. However, considering the findings of previous studies, it is not clear whether targeting the somatotopic organization of M1 corresponding to painful areas can enhance pain relief. In the future, it will be necessary to investigate the optimal stimulation site according to the pain site and to select the optimal target population according to the stimulation site.

Our study has a few limitations. First, the main limitation of this study is the small sample size and the small number of studies included, which made the sensitivity analyses difficult. Second, as a methodological consideration of this analysis, the procedure of meta-analysis must be considered. Although a meta-analysis should be conducted according to a predetermined procedure (Liberati et al., 2009; Rethlefsen et al., 2021), we extracted and analyzed only three RCTs mainly conducted by Osaka University Hospital because there was little data to incorporate. Therefore, there was an obvious selection bias. The findings of this study suggest that a rigorous meta-analysis of the efficacy of rTMS by pain sites needs to be performed, and these findings need to be validated in the future. To achieve this, future prospective clinical trials should also provide site-specific pain results. Third, heterogeneity should be considered when interpreting the results of the meta-analysis. According to the Cochrane Handbook Version 6.2 see text footnote 1, caution should be taken when interpreting heterogeneity due to the poor power of the chi-squared test when the number of studies incorporated in the analysis is small. To compensate for the lack of power, the significance level of heterogeneity for the chi-squared test was set at 0.10 rather than the conventional level of 0.05. In this study, no heterogeneity was identified in the results for upper and lower limb pain, which was the main focus of this study. However, we found moderate heterogeneity in the results of the entire population and facial pain. As far as we could check, through visual inspection of the funnel plots of our previous studies incorporated in these analyses, it does not seem to be a non-specific asymmetry. We believe that the heterogeneity was significant for facial pain (*N* = 16) because the number of patients was too small compared to the analysis of upper limb pain (*N* = 76) and lower limb pain (*N* = 65). Because this study has been analyzed with a small number of patients, with only our previously generated results, it is necessary to take sufficient care while interpreting these results.

## Conclusion

In conclusion, this study suggests differences in the analgesic effects of high-frequency rTMS over the M1 using the figure-of-8 coil for neuropathic pain between pain sites. More importantly, rTMS for upper limb pain was clearly effective in relieving pain. Meanwhile, rTMS for lower limb pain and facial pain did not produce an analgesic effect under the conditions of previous clinical trials. However, considering the small number of included studies, our findings should be considered tentative.

## Data Availability Statement

The raw data supporting the conclusions of this article will be made available by the authors, without undue reservation.

## Ethics Statement

The studies involving human participants were reviewed and approved by the Ethics Committee of Osaka University Hospital. Written informed consent to participate in this study was provided by the participants’ legal guardian/next of kin.

## Author Contributions

KH and NM contributed to trial design and conducted experiments and data collection. YS, SO, and HK supervised the study. All authors contributed to the data interpretation. NM drafted the manuscript. KH edited the manuscript. NM and KH conducted statistical analyses. All authors reviewed and approved the final manuscript.

## Conflict of Interest

The authors declare that the research was conducted in the absence of any commercial or financial relationships that could be construed as a potential conflict of interest.

## Publisher’s Note

All claims expressed in this article are solely those of the authors and do not necessarily represent those of their affiliated organizations, or those of the publisher, the editors and the reviewers. Any product that may be evaluated in this article, or claim that may be made by its manufacturer, is not guaranteed or endorsed by the publisher.

## Footnotes

1
https://training.cochrane.org/handbook/archive/v6.1/chapter-10
