# Supplementary material for: Difference in Analgesic Effects of Repetitive Transcranial Magnetic Stimulation According to the Site of Pain
Source: Front Hum Neurosci. 2021 Nov 26;15:786225. doi: 10.3389/fnhum.2021.786225 (PMC8662379; doi:10.3389/fnhum.2021.786225)
Supplement: Supplementary file 1 [file Table_1.pdf]

Supplementary Table 1 . Characteristics of an RCT of high-frequency rTMS for chronic neuropathic pain (N ≥ 10)

No	Study	Design	N (A/S)	Painful conditions	Stimulation site for				Parameters and Dosage
					Face pain	Upper limbs pain	Lower limbs pain	Hemibody pain	
1	Lefaucheur et al. 2001a	Cross-over 3-ways (10-Hz, 0.5-Hz, sham)	18	mixed central and peripheral	-	M1 hand	-	-	10-Hz, 80%RMT, total 1000 pulses/session, (50 pulses x 20 train/session, 1 session, ITI = 55 sec)
2	Lefaucheur et al. 2001b	Cross-over 2-ways (10-Hz, sham)	14	mixed central and facial	M1 face	M1 hand	-	-	10-Hz, 80%RMT, total 1000 pulses/session, (50 pulses x 20 train/session, 1 session, ITI = 55 sec)
3	Lefaucheur et al. 2004	Cross-over 2-ways (10-Hz, sham)	60	mixed central and peripheral	M1 hand	M1 hand	M1 hand	-	10-Hz, 80%RMT, total 1000 pulses/session, (50 pulses x 20 train/session, 1 session, ITI = 55 sec)
4	Khedr et al. 2005	Parallel sham control	48 (24/24)	mixed central and facial	No details of the painful sites were mentioned. M1 hand contralateral to the painful sites.				20-Hz, 80%RMT, total 2000 pulses/session, (200 pulses x 10 train/session, 5 sessions, ITI = 50 sec)
5	André-Obadia et al. 2006	Cross-over 3-ways (20-Hz, 1-Hz, sham)	12	mixed central and peripheral	M1*	M1*	M1*	M1*	20-Hz, 90%RMT, total 1600 pulses/session, (80 pulses x 20 train/session, 1 session, ITI = 84 sec)
6	Hirayama et al. 2006	Cross-over 5-ways (4 difference stimulation	20	mixed central and peripheral	M1 face + navi S1 + navi PMC + navi	M1 face + navi S1 + navi PMC + navi	M1 face + navi S1 + navi PMC + navi	-	5-Hz, 90%RMT, total 500 pulses/session, (50 pulses x 10

8	Defrin et al. 2007	Parallel sham control	11 (6/5)	post-SCI central	-	-	M1* (Vertex)	-	5-Hz, 115%RMT, total 500 pulses/session, (50 pulses x 500 train/session, 10 sessions, ITI = 30 sec) †
9	Saitoh et al. 2007	Cross-over 4-ways (10-Hz, 5-Hz, 1-Hz, sham)	13	mixed central and peripheral	-	M1 hand + navi	M1 foot + navi	-	5-Hz, 90%RMT, total 500 pulses/session, (50 pulses x 10 train/session, 1 session, ITI = 50 sec)<br>10-Hz, 90%RMT, total 500 pulses/session, (100 pulses x 5 train/session, 1 session, ITI = 50 sec)
10	André-Obadia et al. 2008	Cross-over 3-ways (difference coil orientations, sham)	28	mixed central and peripheral	M1 hand	M1 hand	M1 hand	M1 hand	20-Hz, 90%RMT, total 1600 pulses/session, (80 pulses x 20 train/session, 1 session, ITI = 84 sec)
11	Lefaucheur et al. 2008	Cross-over 3-ways (10-Hz, 1-Hz, sham)	46	mixed central and peripheral	M1 hand	M1 hand	M1 hand	-	10-Hz, 90%RMT, total 1200 pulses/session, (60 pulses x 20 train/session, 1 session, ITI = 54 sec)
12	Kang et al. 2009	Cross-over 2-ways (10-Hz, sham)	11	post-SCI central	-	-	M1 hand	-	10-Hz, 80%RMT, total 1000 pulses/session, (50 pulses x 20 train/session, 5 sessions, ITI = 55 sec)

13	Ahmed et al. 2011	Parallel (quasi-) sham control	27 (17/10)	phantom limb	-	M1 hand	M1 hand	-	20-Hz, 80%RMT, total 2000 pulses/session, (200 pulses x 10 train/session, 5 sessions, ITI = 50 sec)
14	André-Obadia et al. 2011	Cross-over 3-ways (difference of placebo timing)	45	mixed central and peripheral	M1 hand	M1 hand	M1 hand	M1 hand	20-Hz, 90%RMT, total 1600 pulses/session, (80 pulses x 20 train/session, 1 session, ITI = 84 sec)
15	Hosomi et al. 2013	Cross-over 2-ways (5-Hz, sham)	64	mixed central and peripheral	M1 face	M1 hand	M1 foot	-	5-Hz, 90%RMT, total 500 pulses/session, (50 pulses x 10 train/session, 10 sessions, ITI = 50 sec)
16	Jette et al. 2013	Cross-over 3-ways (2 different stimuli sites, sham)	16	post-SCI central	-	M1 hand + navi M1 foot + navi	M1 hand + navi M1 foot + navi	-	10-Hz, 90%RMT, total 2000 pulses/session, (50 pulses x 40 train/session, 1 session, ITI = 25 sec)
17	Onesti et al. 2013	Cross-over sham control	23	diabetic neuropathy	-	-	M1 foot	-	20-Hz, 100%RMT, total 1500 pulses/session, (50 pulses x 30 train/session, 5 sessions, ITI = 30 sec)
18	de Oliveira et al. 2014	Parallel sham control	21 (11/10)	CPSP	Left DLPFC/PMC	Left DLPFC/PMC	Left DLPFC/PMC	Left DLPFC/PMC	10-Hz, 120%RMT, total 1250 pulses/session, (50 pulses x 25 train/session, 10 sessions, ITI = 25 sec)

19	Yılmaz et al. 2014	Parallel sham control	16 (9/7)	post-SCI central	-	-	M1 foot	-	10-Hz, 110%RMT, total 1500 pulses/session, (50 pulses x 30 train/session, 10 sessions, ITI = 25 sec)
20	Attal et al. 2016	Parallel sham control	35 (23/12)	Lumbosacral radiculopathy	-	-	M1 hand	-	10-Hz, 80%RMT, total 3000 pulses/session, (100 pulses x 30 train/session, 3 sessions, ITI = 20 sec)
21	Ayache et al. 2016	Cross-over 3-ways (navi + rTMS, non-navi + rTMS, sham)	66	mixed central and peripheral	M1 hand<br>M1 face + navi	M1 hand<br>M1 face + navi	M1 hand<br>M1 face + navi	-	10-Hz, 90%RMT, total 3000 pulses/session, (100 pulses x 30 train/session, 1 session, ITI = 20 sec)
22	Malavera et al. 2016	Parallel sham control	54 (27/27)	phantom limb	-	-	M1 hand	-	10-Hz, 90%RMT, total 1200 pulses/session, (60 pulses x 20 train/session, 10 sessions, ITI = 54 sec)
23	Nurmikko et al. 2016	Cross-over 3-ways (M1 hotspot, M1 anatomical area, sham (occipital fissure))	38	mixed central, peripheral and others	Site A: M1 hotspot<br>Site B: M1 anatomical area<br>Site C: occipital fissure	Site A: M1 hotspot<br>Site B: M1 anatomical area<br>Site C: occipital fissure	Site A: M1 hotspot<br>Site B: M1 anatomical area<br>Site C: occipital fissure	-	10-Hz, 90%RMT, total 2000 pulses/session, (100 pulses x 20 train/session, 5 sessions, ITI = 60 sec)
24	Nardone et al. 2017	Parallel sham control	12 (6/6)	post-SCI central	Left DLPFC/PMC	Left DLPFC/PMC	Left DLPFC/PMC	Left DLPFC/PMC	10-Hz, 120%RMT, total 1250 pulses/session, (50 pulses x 25 train/session, 10

									sessions, ITI = 25 sec)
25	Shimizu et al. 2017	Cross-over 3-ways (H-coil, Figure-of-8-coil, sham)	18	mixed central and peripheral	-	-	M1 foot (Only during using Figure-of-8 + navi)	-	5-Hz, 90%RMT, total 500 pulses/session, (50 pulses x 10 train/session, 5 sessions, ITI = 50 sec)
26	André-Obadia et al. 2018	Cross-over 3-ways (difference stimulation sites, sham)	32	mixed central and peripheral	M1 hand + navi M1 face + navi	M1 hand + navi M1 face + navi	-	-	20-Hz, 90%RMT, total 1600 pulses/session, (80 pulses x 20 train/session, 1 session, ITI = 84 sec)
27	Choi et al. 2018	Parallel sham control	12 (6/6)	Mild traumatic brain injury	No details of the painful sites were mentioned. M1 hand contralateral to the painful sites.				10-Hz, 90%RMT, total 1000 pulses/session, (50 pulses x 20 train/session, 5 sessions, ITI = 55 sec)
28	Galhardoni et al. 2019	Parallel PSI, ACC, sham	98 (33/33/32)	CPSP and post-SCI central	ACC PSI + navi sham	ACC PSI + navi sham	ACC PSI + navi sham	ACC PSI + navi sham	10-Hz, 90%RMT, total 1500 pulses/session, (100 pulses x 15 train/session, 16 sessions, ITI = 50 sec)

29	Pei et al. 2019	Parallel 5-Hz, 10-Hz, sham	60 (20/20/20)	postherpetic neuralgia	No details of the painful sites were mentioned. M1 hand contralateral to the painful sites.				5-Hz, 80%RMT, total 1500 pulses/session, (5 pulses x 300 train/session, 15 sessions, ITI = 2.5 sec) 10-Hz, 80%RMT, total 1500 pulses/session, (5 pulses x 300 train/session, 15 sessions, ITI = 3 sec)
30	Sun et al. 2019	Parallel sham control	17 (11/6)	post-SCI central	-	Left M1 hand + navi	Left M1 hand + navi	Left M1 hand + navi	10-Hz, 80%RMT, total 1200 pulses/session, (12 pulses x 100 train/session, 6 sessions, ITI = 3 sec)
31	Hosomi et al. 2020	Parallel sham control	142 (72/70)	mixed central and peripheral	M1 face + navi	M1 hand + navi	M1 foot + navi	-	5-Hz, 90%RMT, total 500 pulses/session, (50 pulses x 10 train/session, 5 sessions, ITI = 50 sec)
32	Quesada et al. 2020	Cross-over 2-ways (20-Hz, sham)	42	central	-	M1 hand + navi	M1 hand + navi	M1 hand + navi	20-Hz, 80%RMT, total 1600 pulses/session, (80 pulses x 20 train/session, 4 sessions, ITI = 84 sec)
33	André-Obadia et al. 2021	Cross-over 2-ways (20-Hz, iTBS)	42	mixed central, peripheral and others	M1 hand + navi	M1 hand + navi	M1 hand + navi	M1 hand + navi	20-Hz, 90%RMT, total 1600 pulses/session, (80 pulses x 20 train/session, 5 sessions, ITI = 84 sec)

34	Attal et al. 2021	Parallel M1, DLPFC, sham	149 (49/52/48)	peripheral	M1 hand + navi DLPFC + navi	M1 hand + navi DLPFC + navi	M1 hand + navi DLPFC + navi	M1 hand + navi DLPFC + navi	10-Hz, 80%RMT, total 3000 pulses/session, (100 pulses x 30 train/session, 15 sessions, ITI = 20 sec)
35	Mori et al. 2021b	Cross-over 4-ways (different parameters, sham)	22	mixed central and peripheral	M1 hand + navi	M1 hand + navi	M1 hand + navi	-	10-Hz, 90%RMT, total 2000 pulses/session, (50 pulses x 40 train/session, 1 session, ITI = 25 sec)
36	Ojala et al. 2021	Cross-over 3-ways (M1, sham, S2)	21	CPSP	-	M1 + navi or S2 + navi	-	-	10-Hz, 90%RMT, total 5050 pulses/session, (101 pulses x 50 train/session, 10 sessions, ITI = 50 sec)

The types of painful conditions were categorized as central neuropathic pain, peripheral neuropathic pain, facial pain, and others.

A, active rTMS group; S, sham rTMS group; CPSP, Central Post-Stroke Pain; MS, Multiple sclerosis; SCI, Spinal Cord Injury; CRPS, Complex Regional Pain Syndrome; M1 hand (face, foot) Primary motor cortex of hand (face, foot) contralateral to the painful site; M1*, no mention the detailed site of coil; PMC, Premotor Cortex; SMA, Supplementary Motor Area; S1, Primary Somatosensory Cortex; S2, Secondary Somatosensory Cortex; DLPFC, Dorsolateral Prefrontal Cortex; ACC, Anterior Cingulate Cortex; PSI, Posterior Superior Insula; navi, navigation-guided rTMS during each session; RMT: Resting Motor Threshold; ITI, Inter-Train Interval; sec, second.

† Inconsistency between the number of trains and the number of pulses/sessions in the reported parameters.
